# Is the number of prescriptions an appropriate metric for outpatient antimicrobial consumption? A comparison between the prescription counts and days supplied

**DOI:** 10.1017/ice.2022.189

**Published:** 2023-06

**Authors:** Satoshi Kakiuchi, Eli N. Perencevich, Daniel J. Livorsi, Michihiko Goto

**Affiliations:** 1 Center for Access & Delivery Research and Evaluation (CADRE), Iowa City Veterans’ Affairs Health Care System, Iowa City, Iowa; 2 University of Iowa, Department of Internal Medicine, Iowa City, Iowa

## Abstract

The optimal metric for outpatient antimicrobial stewardship has not been well defined. The number of antibiotic prescriptions per clinic visit does not account for the therapeutic duration. We found only moderate association between prescription-based metrics and days-supplied–based metrics. Outpatient antibiotic consumption metrics should incorporate the duration of therapy.

Monitoring antibiotic usage is critical for promoting the appropriate use of antibiotics. Ambulatory antibiotic prescriptions reached 251.1 million in 2019.^1^ However, defining appropriate metrics for outpatient antibiotic consumption is challenging. The Society of Infectious Diseases Pharmacists (SIDP) suggests tracking prescription records.^2^ The Centers for Disease Control and Prevention (CDC) Core Elements of Antimicrobial Stewardship guideline recommends that every outpatient setting track the percentage of all visits leading to antimicrobial prescriptions.^3^

These recommendations are constrained by data availability. The duration of therapy is not a required component in prescriptions and often requires calculations from the total quantity and the direction of use. Also, the presence of a prescription does not necessarily mean the medication is dispensed from pharmacies.^4,5^ The information for medication dispensing at pharmacies may not be immediately transmitted to providers, whereas the number of prescriptions can be more easily obtained through electronic medical record (EMR) systems. In addition, outpatient antibiotic prescriptions are often given for an extended period at a single visit (typically for several days, but up to 90 days), and a single prescription may account for multiple dispensings from pharmacies if refills are included. Therefore, the number of antibiotic prescriptions may not accurately reflect the amount of antibiotics dispensed, especially for prescriptions with longer durations. These limitations can lead to inappropriate assessment of trends within the same system or for interfacility comparison.

From pharmacies at the Veterans’ Health Administration (VHA), which operates both outpatient care deliveries and pharmacies, we compared 2 metrics for outpatient antibiotic consumption: the number of prescriptions in EMRs and the number of dispensed days, or days supplied.

## Methods

### Study design

We conducted a retrospective study of all patients who received antibiotic prescriptions at outpatient clinics through in-person or video telehealth visits within 129 VHA healthcare systems filled at VHA pharmacies from January 2010 to December 2019. We obtained data from the VHA Corporate Data Warehouse, and we counted antibiotic prescriptions and days supplied (as specified by prescribers) from VHA pharmacies. We included antibiotics in the National Healthcare Safety Network (NHSN) protocol^6^ and only systemic oral agents prescribed on weekdays during business hours. Data for antibiotic prescriptions filled outside the VHA were not available for analysis. We also excluded outpatient prescriptions between 2 days before and 1 day after hospital discharge as discharge prescriptions to avoid interfacility bias between facilities with inpatient care services and those without.

### Statistical analyses

To assess differences in longitudinal trends for the 2 metrics, we calculated the VHA system-wide number of prescriptions and days supplied per clinic visit for each year and plotted a line graph showing how the prescription number and days supplied changed from 2010. To assess the impact of differences on interfacility comparisons, we calculated the number of prescriptions and days supplied per clinic visit for each healthcare system and ranked facilities using each of the 2 metrics. We then compared rankings using the Kendall *τ*_*B*_ coefficient. We interpreted |*τ*_*B*_| ≥ 0.7 as strong correlations, 0.5 ≤ |*τ*_*B*_| < 0.7 as moderate correlations, and |*τ*_*B*_| < 0.5 as weak correlations. All statistical analyses were performed using R version 4.1.2 software (R Foundation for Statistical Computing, Vienna, Austria).

### Ethics

The Institutional Review Board of the University of Iowa and the Research and Development Committee at Iowa City Veterans’ Health Care System approved this study with a waiver of informed consent.

## Results

During the study period, 13,373,460 clinic visits (5.0%) of the 265,613,607 total clinic visits had an antibiotic prescription, and the total of days supplied was 182,793,572. Clinic visits, prescriptions, days supplied, and mean days supplied are shown in Supplementary Table 1 (online). The mean days supplied per prescription remained largely unchanged during the study period.

**Fig. 1.**
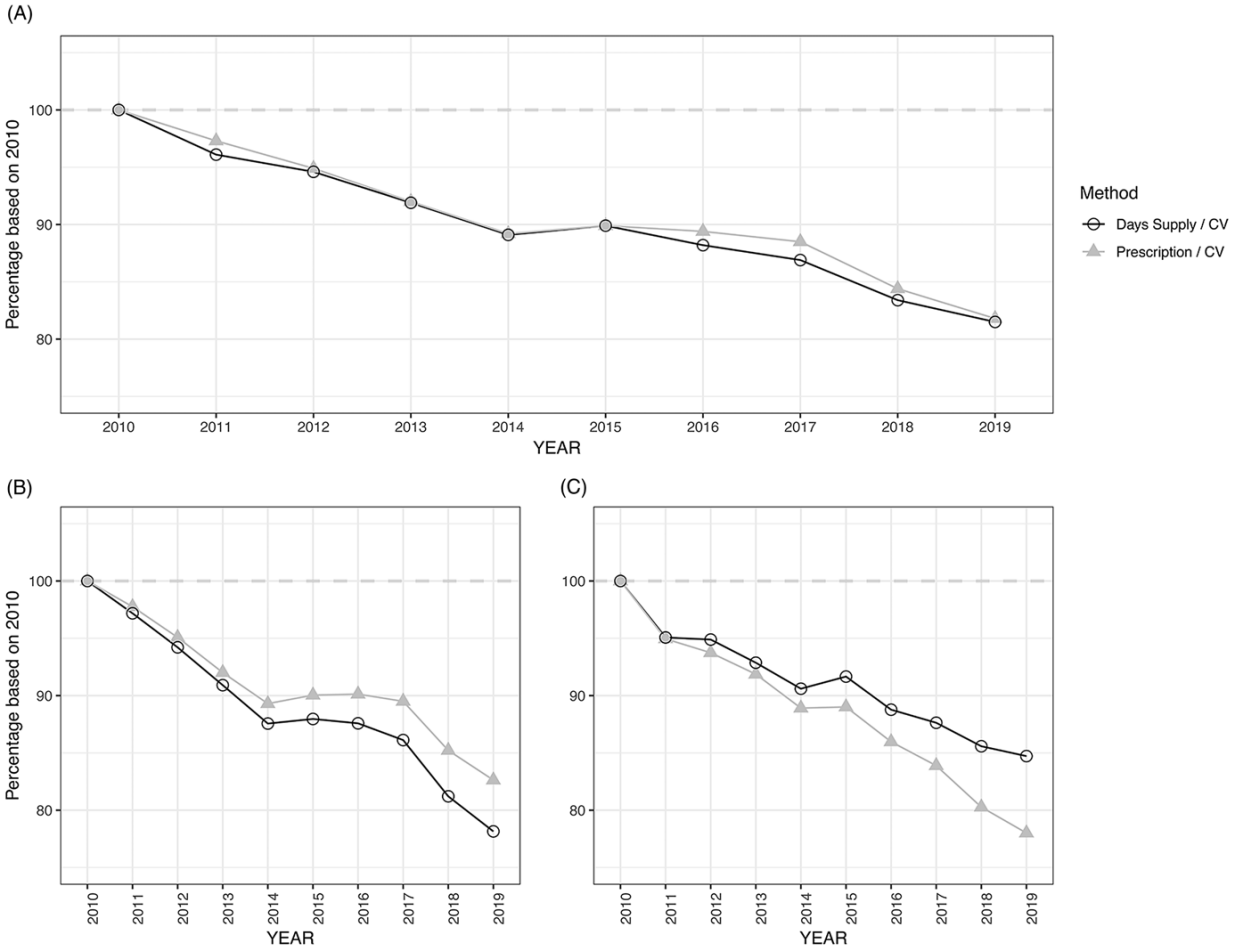
Percentage change in the number of outpatient antimicrobial prescriptions and days supplied per clinic visit across 129 VHA medical centers, 2010–2019. (A) Trends in total number of prescriptions and days supplied per clinic visit (CV). (B) Trends of total number of prescriptions and days supplied, when limited to short-term therapy (≤14 days). (C) Trends of total number of prescriptions and days supplied, when limited to long-term therapy (>14 days). Grey dashed line represents 100% (same level as 2010).

Figure 1 shows proportional changes in the number of prescriptions and days supplied, using 2010 data as the baseline. Comparing the number of prescriptions and days supplied normalized by the number of clinic visits, these 2 metrics changed in parallel, with only minor differences from 2010 to 2019: −18.2% in the number of prescriptions and −18.5% in days supplied (Fig. 1A). However, when we focused on short-term prescriptions typically used for acute illnesses (≤14 days), prescription number and days supplied showed an increasing discrepancy. The number of prescriptions underestimated the decline compared to days supplied: −17.4% and −21.9%, respectively (Fig. 1B). On the other hand, long-term prescriptions (≥15 days) showed the opposite discrepancy: −15.3% and –22.0%, respectively (Fig. 1C).

**Fig. 2.**
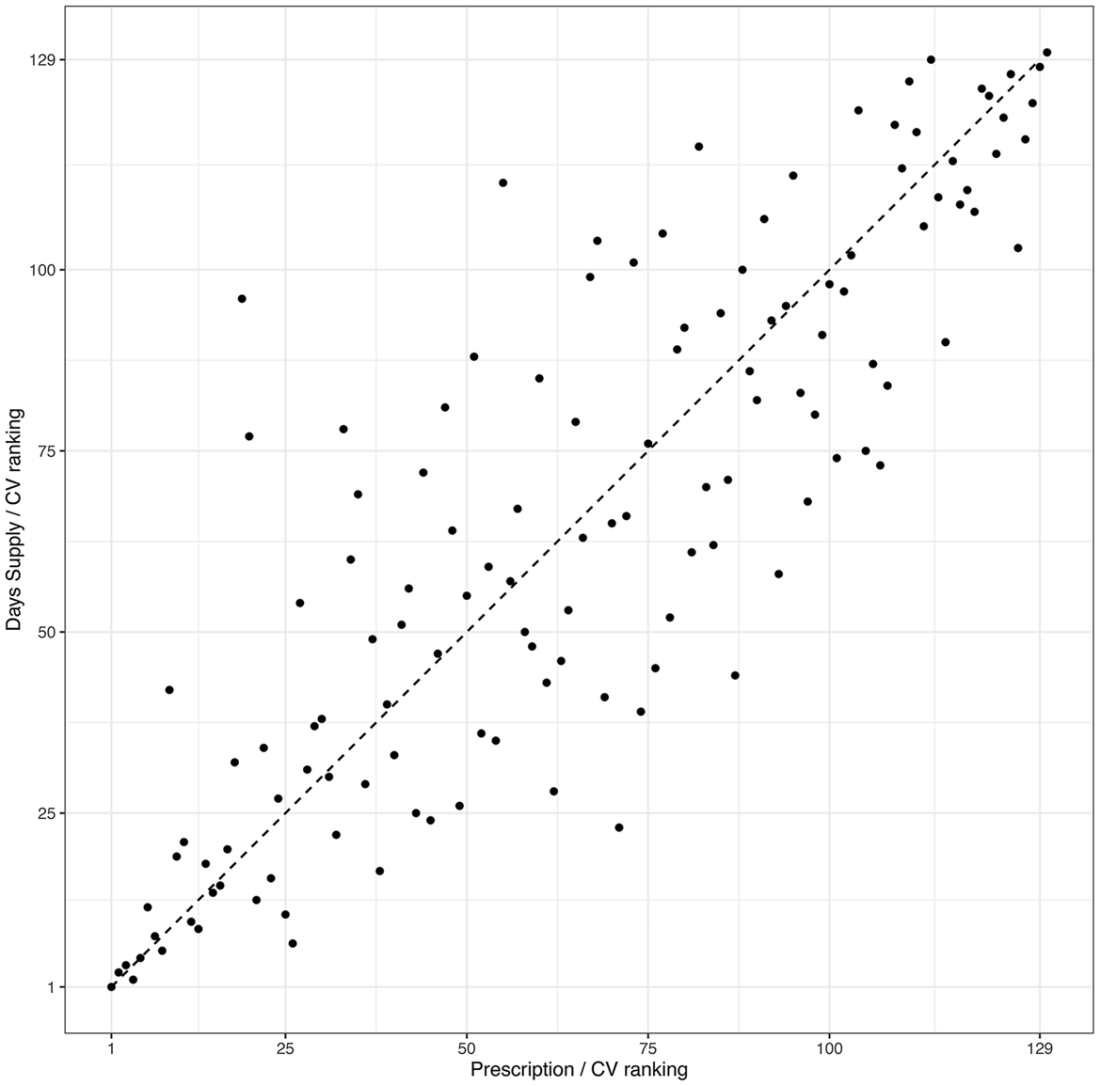
Ranking difference between the number of prescriptions and days supplied per clinic visit (CV) of 129 Veterans’ Healthcare Administration in 2019. Dashed line: same ranking in the number of prescriptions and days supplied per clinic visit.

For interfacility comparisons, we detected substantial differences in rankings of healthcare systems based on the 2 metrics (Fig. 2). The *τ*_*B*_ coefficient was 0.68 (95% confidence interval, 0.61–0.74), indicating only moderate correlations between interfacility comparisons based on 2 metrics.

## Discussion

In this 10-year review of outpatient antibiotic prescriptions across 129 VHA hospitals, we found that the number of prescriptions may not accurately reflect antibiotic consumption in outpatient settings. This discrepancy is likely driven by a wide range of intended therapeutic durations for various infectious diseases. Unlike prescriptions for the management of chronic health conditions, a substantial proportion of antibiotic prescriptions are short-course therapies, and the days supplied per clinic visit ranges widely.^7^ Using days supplied per clinic visit as a metric may overcome this limitation and encourage shorter durations of therapy, which are increasingly supported by the literature.

On the other hand, identifying the number of prescriptions has an obvious advantage. These numbers can be obtained more easily from EMRs without relying on pharmacies that may or may not be affiliated with facilities from which a prescription is derived and without need for calculating duration of therapy. These data are particularly useful when most prescriptions are filled at community pharmacies because most do not automatically transmit data to prescribing institutions. Bidirectional data transmission between prescribing facilities and community pharmacies has many benefits beyond just data accuracy, and interconnectivity must be improved before days supplied per clinic visit can be used more widely as a metric.

Our study has several limitations. First, no information regarding patient adherence was available. However, at least days supplied accurately reflects the amount of antibiotics released from healthcare systems into communities, and patients or families may use unused antibiotics outside of intended purposes. Second, we could include only prescriptions filled and dispensed at VHA pharmacies in the analysis, and this may not reflect the entire picture of the outpatient antimicrobial consumption in the VHA system. Therefore, we excluded prescriptions written on weekends and outside business hours to minimize bias due to the use of non-VHA community pharmacies. Third, we used the days supplied specified by prescribers which might be inaccurate if the antibiotics were used intermittently. In our data set, this occurred in ∼6% of all prescriptions (when azithromycin was excluded, ∼3%). Lastly, the VHA provides services to more elderly populations with no pediatric care, which may limit the generalizability of our findings to non-VHA populations.

We have demonstrated that the number of antibiotic prescriptions may be useful only when assessing the overall trend within a healthcare system. It may not provide accurate estimates when used for interfacility comparisons for antibiotic consumption or trends for the management of acute infections (short-course therapy) or long-term therapy. As the interoperability and data transmissibility of EMR systems improve, traditional but suboptimal metrics should be replaced by metric(s) that better reflect dispensed amounts, whenever feasible, to provide a higher content validity. Additionally, metrics that capture the duration of therapy may encourage prescriptions with shorter durations and lessen the ecological impact of outpatient antimicrobial use.

## Supplementary material

For supplementary material accompanying this paper visit https://doi.org/10.1017/ice.2022.189. click here to view supplementary material
