# Supplementary material for: Is the number of prescriptions an appropriate metric for outpatient antimicrobial consumption? A comparison between the prescription counts and days supplied
Source: Infect Control Hosp Epidemiol. 2022 Aug 23;44(6):941–4. doi: 10.1017/ice.2022.189 (PMC10262164; doi:10.1017/ice.2022.189)
Supplement: Supplementary file 1 [file S0899823X22001891sup001.docx]

**Supplementary Table.** Number of outpatient visits, prescriptions, days supply, and mean days supply, 2010-2019.

|  | 2010 | 2011 | 2012 | 2013 | 2014 | 2015 | 2016 | 2017 | 2018 | 2019 |
| --- | --- | --- | --- | --- | --- | --- | --- | --- | --- | --- |
| Outpatient visits | 23,956,392 | 24,568,604 | 25,037,015 | 25,457,832 | 26,269,486 | 26,990,705 | 27,525,471 | 27,907,496 | 28,568,230 | 29,332,376 |
| Prescriptions | 1,334,060 | 1,330,955 | 1,322,481 | 1,304,024 | 1,305,380 | 1,350,711 | 1,370,626 | 1,376,083 | 1,342,324 | 1,336,816 |
| Days supply (total) | 18,355,430 | 18,090,593 | 18,139,216 | 17,927,735 | 17,936,694 | 18,582,528 | 18,599,136 | 18,579,798 | 18,264,335 | 18,318,107 |
| Days supply (mean) | 13.8 | 13.6 | 13.7 | 13.7 | 13.7 | 13.8 | 13.6 | 13.5 | 13.6 | 13.7 |

Days supply (mean) were calculated by days supply/prescriptions
